# Advances in direct optical detection

**DOI:** 10.1007/s00216-020-02617-5

**Published:** 2020-05-08

**Authors:** Antje J. Baeumner, Guenter Gauglitz, Jiří Homola

**Affiliations:** 1 Institute for Analytical Chemistry, Bio- and Chemosensors, University of Regensburg, Universitätsstraße 31, 93053 Regensburg, Germany; 2 Institute for Theoretical and Physical Chemistry, Eberhard-Karls-University, Auf der Morgenstelle 18, 72076 Tübingen, Germany; 3 Institute of Photonics and Electronics of the Czech Academy of Sciences, Chaberska 57, 18251 Prague 8, Czech Republic

Today’s societal grand challenges drive the need for new sensing principles, new sensors, and entirely new analytical solutions as never before. Global challenges, such as climate change, infectious diseases and their migration, air and ocean pollution, food and water safety, and security, will require our attention for decades to come. The Food and Agriculture Organization of the United Nations (FAO) raises the question of how to feed 10 billion people in 2050 which not only calls for a needed dramatic increase in available safe food and its hopefully sustainable agricultural production strategies, but also relates to our ability to monitor conditions required to achieve this challenge. The Covid-19 pandemic of 2020 caused by Coronavirus SARS-CoV-2 reminded all of us of the urgent need for rapid point-of-care diagnostics, the need that exists also for many other health conditions. New sensors for the monitoring of patients at their bedsides or at home are envisioned to be important technologies that will support personalized medicine, lead to reductions of the health care costs, and enable health care in resource-limited settings. The increasingly global dimension in the food supply chain raises the need for sensing technologies to ensure food quality and safety and to combat food fraud. Although analytical chemistry has solutions for many of these challenges today, they often require costly and bulky laboratory equipment not suited for rapid analysis in the field.

Using light for sensing is a century-old principle, which, combined with advances in physics, engineering, materials research, and (bio) chemistry, offers a myriad of strategies to address current and future analytical challenges. The main drivers of current sensor research include the needs for the detection of analytes at very low levels in complex real-world samples, robustness for applications in the field, and the ability to parallelize assays for multiparameter and multianalyte measurements. As this topical collection demonstrates, current optical sensors use a multitude of optical methods (spectroscopies, scattering, interferometry, surface plasmon resonance, luminescence) and platforms (optical waveguides and fibers, resonators, etc.) which employ electromagnetic radiation across a broad range of wavelengths (from UV and Vis, through IR to THz). Their combination with selective coatings and microfluidic devices is vital for the development of analytical systems and has been gaining much interest. The five critical reviews collected in this special issue highlight centrally important lines of research in direct optical sensing, also including emerging areas, such as droplet-based approaches, micro-optofluidics, and enhanced sensing through advanced nanostructures. The research papers discuss new developments in sensing methods and applications that range from biochemical and cell-based studies to medical diagnostics and environmental monitoring. Emphasis of this topical collection is on label-free approaches for direct optical detection. They are well suited not only for on-site detection due to shorter assay times and simple assay protocols, but also for the real-time measurement of biomolecular interactions. As the bio (medical) community is increasingly interested in antibody identification, target screening, protein-protein interactions, and immunological screening, such label-free direct optical sensing approaches can in fact provide a platform for the necessary high-throughput screening.

We would like to thank all of the authors for their interesting, high-quality, and timely contributions. We are also grateful to all of the reviewers whose constructive criticisms and thoughtful suggestions helped to ensure that the papers accepted for this special issue of *ABC* meet the highest scientific standards. We hope that this topical collection will become a useful source of information for sensor scientists and help advance the field of direct optical sensing in a world full of opportunities and challenges.

